# Associations between nocturnal sleep duration, midday nap duration and body composition among adults in Southwest China

**DOI:** 10.1371/journal.pone.0223665

**Published:** 2019-10-16

**Authors:** Mengxue Chen, Xiao Zhang, Yi Liang, Hongmei Xue, Yunhui Gong, Jingyuan Xiong, Fang He, Yanfang Yang, Guo Cheng

**Affiliations:** 1 West China School of Public Health and Healthy Food Evaluation Research Center, Sichuan University, Chengdu, P.R. China; 2 College of Public Health, Hebei University, Baoding, P. R. China; 3 Department of Obstetrics and Gynecology, West China Second University Hospital, Sichuan University, Key Laboratory of Birth Defects and Related Diseases of Women and Children (Sichuan University) of Ministry of Education, Chengdu, P. R. China; 4 Department of Obstetrics and Gynecology, Longquanyi District of Chengdu Maternity & Child Health Care Hospital, Chengdu, P. R. China; 5 West China School of Public Health and Healthy Food Evaluation Research Center and State Key Laboratory of Biotherapy and Cancer Center, Sichuan University, Chengdu, P.R. China; Kasturba Medical College Mangalore, INDIA

## Abstract

**Objective:**

We aim to explore the relationship between nocturnal sleep duration (NSD) and midday nap duration (MND) with body composition among Southwest Chinese adults.

**Methods:**

Data on sleep duration of 3145 adults in Southwest China (59.4% women) were obtained between 2014 and 2015 through questionnaires. Height, weight, and waist circumference (WC) were measured to calculate body composition (body mass index (BMI), percentage of body fat (%BF), and fat mass index (FMI)). Linear regression models were used to assess gender-specific associations between NSD and body composition. The relationship between MND with the odds of overweight and central obesity has been evaluated by logistic regression models.

**Results:**

NSD has the inverse relation with males’ BMI, WC, %BF and FMI after adjusting for all covariates (all P <0.0007), exclusive of females’ (all P >0.4). After adjustment for potential confounders, compared to the subjects in the no midday nap group, the subjects who napped 0.1–1 hour were independently associated with a less prevalence of overweight in both women (OR: 0.72, 95%CI: 0.55–0.95) and men (OR: 0.71, 95%CI: 0.52–0.98). MND was not associated with central obesity.

**Conclusions:**

Among Southwest Chinese adults, lower NSD might be related to higher BMI, WC, %BF and FMI among men. Additionally, MND is associated with overweight in adults.

## Introduction

Obesity has become a worldwide epidemic. Overweight and obesity are suggested to be risk factors of many chronic diseases such as cancer [1] and diabetes mellitus [2], increased body weight is also associated with higher risk of cardiovascular morbidity [3]. With the development of economy and society, obesity has become a major public health problem in China: according to the data of China Health and Nutrition Survey, 42.3% of Chinese adults were overweight in 2011, and 11.3% were obese [4].

In modern industrial society, sleep deprivation in general people has become an important public health concern [5]. Previous studies among Western adults indicated that short nocturnal sleep duration (NSD) was associated with higher body mass index (BMI) [6–8]. A meta-analysis has reported that sleep duration was also associated with elevated waist circumference (WC) in adults [9]. The relation between short NSD and higher body fatness was shown in Greek women [10]. In a survey with 0.5 million Chinese adults, the rate of insufficient sleep was 23.1% [11]. However, the research on NSD and body composition among Chinese adults is scarce.

Several studies have examined the role of gender on the relationship between sleep duration and body composition. Some of them have found stronger associations among males[12,13] or females[14,15], and others have shown no gender difference[6]. Given the conflicting results of possible gender differences in the literature, and the relationship between sleep duration and BF, FMI was also not explored in these studies, we are particularly interested in studying gender-specific associations between sleep duration and body composition.

Compared with other countries, the midday nap (siesta) is very common in China. The traditional Chinese view is that it can supplement the lack of sleep at night and be beneficial to physical and mental health [16]. In a large Mediterranean cohort study, the researchers observed that short siesta might be an independent protective factor for obesity among Spanish adults [17]. But there is little epidemiological evidence about the relationship between midday nap and body composition in Chinese adults.

Therefore, we aimed to analyze the relevance of NSD and MND for body composition, indicated by BMI, WC, percentage of body fat (%BF), and fat mass index (FMI) in both females and males by using the baseline data of Nutrition and Health in Southwest China (NHSC) study.

## Materials and methods

### Study sample

We used data from the baseline survey of the NHSC study, which is a multicenter, population-based ongoing prospective study that aim to investigate the impact of nutritional factors and lifestyle factors on the development of obesity, diabetes, CVD and changes in the quality of life. Until 2017, NHSC recruited participants from four Chinese field centers (Chengdu, Chongqing, Kunming and Guiyang), included 29 study sites (10 communities and 19 villages). Details on the design and methods of the NHSC study have been previously described [18].

The participants were invited to the study centers for interviews. The survey included anthropometric measurements, questionnaires and face-to-face interviews by trained investigators about dietary information, lifestyles and socioeconomic status. Participants were excluded with the following conditions or diseases: a) if they had major organ diseases, including heart, liver and kidney disease; b) if they had mental diseases; c) if they were taking hormone-based drugs or other medicines that affect blood glucose and lipids; d) if they were pregnant or lactating women. The study was approved by the Ethics Committee of Sichuan University. All the respondents had signed informed consent.

This analysis used the data of 3336 adults from 2014 to 2015. Of these, 201 adults were excluded: 52 had missing sleep data, 37 had incomplete anthropometric data, 89 had illogical energy intakes reported in 24-hour recalls (< 800 or > 4200 kcal/d for men and < 500 or > 3500 kcal/d for women) [19] and 23 had incomplete information on potential confounders. This resulted in a final sample of 3135 adults (59.4% women).

### Sleep information

Information on sleep was collected in face-to-face interviews by trained interviewers. Nocturnal sleep duration (NSD) was derived from the answer to the question, “How many hours per day do you sleep at night during the weekdays and during the weekends?” An overall average NSD was calculated as: (weekday duration × 5 + weekend duration × 2)/7. Because the National Sleep Foundation suggests that adults should sleep 7~9 hours per night [20], we created the following three groups of NSD: < 7 hours, 7~9 hours, and > 9 hours per night. Habitual midday nappers were defined as those who had taken a planned or regular nap as a habit more than three times per week after lunch over the past twelve months. Midday nap duration (MND) was assessed by asking “Do you have a midday napping habit after lunch?” Individuals who reported “Yes” were further asked about the average duration of their naps. We categorized MND as follows: none (0 hour), moderate (0.1~1 hour), and high (> 1 hour) [21].

### Anthropometry

Data of anthropometric measurements included height, weight and WC. All measuring tools were calibrated before measurements. The trained investigator measured the height and weight by ultrasonic electronic meter (Weight and Height Instrument DHM-30; Dingheng Ltd., Zhengzhou Province, China). Height was assessed to the nearest 0.1 cm and weight was measured to the nearest 0.1kg. WC was measured to the nearest 0.1 cm using a non-elastic tape at a point midway between the lowest rib margin and the iliac crest in a horizontal plane. Height, weight and WC were measured two times and final values were derived from the mean of two measurements. BMI was calculated as weight (kg) divided by height squared (m^2^). We defined individuals as overweight according to the World Health Organization (WHO) cut-offs for adults[22] and central obesity according to the International Diabetes Federation (IDF)[23]. %BF was calculated using the equations from Liu, X. et al. [24]. FMI were calculated according to the formula [25]: (weight (kg) × %BF)/height (m^2^).

### Energy intake (EI)

Dietary data were collected in face-to-face interviews via three 24-hour dietary recall. Participants were asked to recall all the details on recipes, brands and types of food items they consumed and the corresponding timing. Prototypes of standard serving bowls, plates and cups were showed to the respondents to ensure the accuracy of the estimated portion sizes. Using the continuously updated in-house nutrient database [26], dietary intake data from the 24-hour recalls were converted to energy and nutrient data through NCCW software (version 11.0; Qingdao University Medical College, Shandong Province, China). The database in NCCW reflects the Chinese Foods Composition [27]. Final EI was the average of the three 24-hour recalls.

### Additional information

Demographic data such as sex, age (years), marital status (single or married), educational attainment (12 or more years of schooling; yes/no) and dietary behavior were collected through interviewer-administered questionnaires in the face-to-face interview.

The physical activity questionnaire was based on the validated questionnaire [28]. The questionnaire was administered and responses recorded in the face-to-face interview. To collect information about usual type and duration of activities related to work, household chores, and leisure-time exercise inside and outside the workplace during the past year, our questionnaire was designed to include a checklist of 38 items, and used a 2011 update of a published compendium of physical activity by category [29] to determine the metabolic equivalent task value of each physical activity. Moderate to vigorous physical activity (MVPA) was characterized as greater than or equal to 3 metabolic equivalent tasks. Energy expended on MVPA per week was calculated.

### Statistical analyses

SAS procedures (version 9.4, SAS Inc, Cary, NC) were used for all data analyses. All analyses were performed with a significance level at two-sided P < 0.05. Normality of all continuous variables was examined by using normal probability plots and the Kolmogorov-Smirnov test. All continuous variables were non-normality (all P <0.03). Continuous variables were presented as median (25th percentile, 75th percentile). There were significant differences in body composition between different genders, and the gender distribution was imbalanced in present study. As a result, the analyses in this study was performed for females and males separately.

To investigate the relevance of NSD for body composition, multivariable generalized linear models (PROC GLM in SAS) were performed. NSD was defined as the independent variable. Body composition including BMI, WC, %BF and FMI were dependent variables in separate models. To improve the fitting effect of the models, log-transformed values of BMI, WC, %BF and FMI were used in the models.

In the basic models, NSD were the independent predictors. The following variables may potentially affect these associations were considered: gender, age (years), marital status (single or married), educational level (12 or more years of schooling; yes/no), MND, MVPA (MET-hour/week) and EI (kcal/d). Each potential confounder was initially considered separately and included if it substantially modified the association of sleep duration with body composition. Thus, age, marital status and educational level were retained in model 1. In model 2, we controlled for sleep confounding by adding MND. In a final model, we performed further adjustments for the possible effects of energy balance by adding EI and MVPA (model 3). The adjusted means were the least-squares means predicted by the model when the other variables were held at their mean values.

Previous studies have shown that the relationship between MND and body composition is not linear. Associations between the MND and the odds for overweight and central obesity in our study were tested using logistic regression models (PROC LOGISTIC in SAS). We also constructed three models, including age, marital status, educational level, NSD, MVPA and EI as confounders.

## Results

Participants who were excluded from the study sample (n = 201) did not differ in age, gender, marital status and educational status from those who were included (n = 3135) (all P >0.7, data not shown).

**Table 1**shows subject characteristics by groups of gender. A total of 1274 men and 1861 women were included in the main analyses. About 59.4% of our participants were women. Women included in the present analysis had a mean age of 56.1 years. Men were aged at 45.3 years. Compared to women, men have higher BMI and WC, but lower BF and FMI.

**Table 1:** Characteristics by participants of gender (n = 3135).

Characteristics	Female	Male	P value
n (%)	1861 (59.4)	1274 (40.6)	
Age (years)	56.1 (46.4, 61.6)	45.3 (31.7, 59.0)	<0.0001
High education level^a^ (n (%))	526 (28.3)	616 (48.4)	<0.0001
Married (n (%))	1508 (81.0)	1044 (81.9)	0.7
MVPA^b^ (MET-h/week)	15.9 (10.2, 22.5)	10.5 (43.1, 15.6)	<0.0001
Energy intake (kcal/d)	1712.1 (1458.6, 2031.7)	2068.9 (1737.4, 2468.0)	<0.0001
MND (none^c^, n (%))	670 (36.0)	437 (34.3)	0.6
NSD (7–9 h/night, n (%))	1148 (61.7)	795 (62.4)	0.03
Body mass index (kg/m^2^)	23.4 (21.3, 25.7)	24.3 (22.2, 26.3)	<0.0001
Waist circumference (cm)	84.3 (77.6, 90.8)	86.98 (81.1, 92.6)	<0.0001
Percentage body fat (%)	33.7 (31.0, 35.9)	22.6 (19.9, 24.6)	<0.0001
Fat mass index (kg/m^2^)	7.9 (6.7, 9.2)	5.5 (4.4, 6.4)	<0.0001
Overweight^d^	603 (32.4)	526 (41.3)	<0.0001
Central obesity^e^	1249 (67.1)	473 (37.1)	<0.0001

Values are median (25th percentile, 75th percentile) or frequencies. Test for difference between males and females was performed by using Wilcoxon rank-sum for non-normally distributed continuous variables and chi-square test for categorical variables.

MVPA = Moderate to Vigorous Physical Activity; MND = Midday Nap Duration; NSD = Nocturnal Sleep Duration.

^a^ School education at least 12 years.

^b^ MVPA energy expenditure, energy expended on moderate-to-vigorous physical activities (MET-hour/week) [29].

^c^ 0 hour/d midday nap duration.

^d^ Overweight defined according to the WHO cut-offs for adults[22], Body mass index ≥25kg/m^2^.

^e^ Central obesity defined according to the IDF[23], Waist circumference ≥80 cm for women, ≥90 cm for men.

Characteristics of females and males according to groups of NSD are presented in Table 2. There was no significant difference in BMI, WC, BF and FMI among three female groups. Men with the shortest NSD had a significantly higher percentage of high education level, BMI, WC, BF and FMI; less of them hadn’t taken midday nap. Table 3 shows the characteristics of women and men grouped by MND. Adults who take 0.1-1h midday nap had lowest BMI, WC, BF and FMI in both women and men.

**Table 2:** Characteristics by groups of NSD (n = 3135).

	Female			P value	Male			P value
	NSD^a^ (h/night)				NSD (h/night)			
	7	7~9	> 9		7	7~9	> 9	
**N**	574	1148	139		345	795	134	
**Age (years)**	58.0 (49.0, 62.3)	55.1 (45.4, 61.3)	51.6 (43.4, 58.6)	<0.0001	48.3 (35.8, 59.5)	43.9 (30.7, 59.0)	42.1 (30.9, 57.0)	0.05
**High education level**^**b**^ **(n (%))**	147 (25.6)	352 (30.7)	27 (19.4)	0.05	191 (55.4)	387 (48.7)	38 (28.4)	0.0003
**Married (n (%))**	461 (80.3)	926 (80.7)	121 (87.1)	0.3	293 (84.8)	636 (80.0)	115 (85.8)	0.2
**MVPA**^**c**^ **(MET-h/week)**	16.1 (10.5, 22.4)	16.0 (10.0, 22.7)	13.0 (8.0, 22.5)	0.3	9.6 (6.0, 15.9)	10.8 (6.5, 17.3)	9.9 (4.9, 15.4)	0.1
**Energy Intake (kcal/d)**	1705.7 (1451.5, 2057.2)	1722.9 (1462.5, 2018.8)	1720.1 (1407.7, 2035.3)	0.9	2017.1 (1767.4, 2436.9)	2119.52 (1730.1, 2489.2)	2003.9 (1717.9, 2364.8)	0.3
**MND (none**^**d**^**, n (%))**	197 (34.3)	414 (36.1)	59 (42.4)	0.1	96 (27.8)	276 (34.7)	65 (48.5)	0.001
**BMI (kg/m**^**2**^**)**	23.4 (21.5, 25.7)	23.4 (21.3, 25.8)	22.8 (21.1, 25.3)	0.3	24.5 (22.7, 26.7)	24.3 (22.1, 26.4)	23.3 (21.0, 25.8)	0.007
**WC (cm)**	84.6 (78.6, 91.4)	84.3 (77.5, 90.9)	82.9 (76.3, 89.1)	0.3	88.3 (83.1, 93.0)	87.0 (80.9, 92.9)	84.2 (78.1, 90.3)	0.0008
**BF (%)**	34.0 (31.5, 36.1)	33.6 (31.0, 35.9)	32.8 (30.1, 35.4)	0.07	23.0 (20.7, 24.7)	22.6 (19.8, 24.7)	21.1 (18.3, 23.8)	0.001
**FMI (kg/m**^**2**^**)**	8.0 (6.8, 9.3)	7.9 (6.6, 9.2)	7.4 (6.4, 9.0)	0.2	5.7 (4.7, 6.4)	5.5 (4.4, 6.5)	4.9 (3.9, 6.1)	0.002
**Overweight**^**e**^	180 (31.4)	382 (33.3)	40 (28.8)	0.6	149 (43.2)	335 (42.1)	42 (31.3)	0.1
**Central obesity**^**f**^	395 (68.8)	769 (67.0)	85 (61.1)	0.4	143 (41.4)	295 (37.1)	35 (26.1)	0.05

Values are median (25th percentile, 75th percentile) or frequencies. Test for difference between groups of NSD was performed by using Wilcoxon rank-sum tests for non-normally distributed continuous variables, chi-square test for categorical variables.

NSD = Nocturnal Sleep Duration; MVPA = Moderate to Vigorous Physical Activity; MND = Midday Nap Duration; BMI = Body Mass Index; WC = Waist Circumference; BF = Percentage Body fat; FMI = Fat Mass Index.

^a^ NSD was grouped according to the National Sleep Foundation [20].

^b^ School education at least 12 years.

^c^ MVPA energy expenditure, energy expended on moderate-to-vigorous physical activities (MET-hour/week) [29].

^d^ 0 hour/d midday nap duration.

^e^ Overweight defined according to the WHO cut-offs for adults[22], BMI ≥25kg/m^2^.

^f^ Central obesity defined according to the IDF[23], WC ≥80 cm for women, ≥90 cm for men.

**Table 3:** Characteristics by groups of MND (n = 3135).

	Female			P value	Male			P value
	MND^a^ (h/night)				MND (h/night)			
	0	0.1~1	> 1		0	0.1~1	> 1	
**n**	671	978	212		436	680	158	
**Age (years)**	57.4 (48.8, 61.6)	55.3 (45.5, 61.5)	52.4 (43.5, 61.2)	0.03	45.5 (33.2, 57.8)	44.3 (30.6, 59.0)	46.6 (32.9, 60.6)	0.2
**High education level**^**b**^ **(n (%))**	152 (22.7)	334 (34.2)	40 (18.9)	<0.0001	196 (45.0)	363 (53.4)	58 (36.7)	0.02
**Married (n (%))**	560 (83.5)	771 (78.8)	1179 (84.4)	0.1	360 (82.6)	549 (80.7)	134 (84.8)	0.6
**MVPA**^**c**^ **(MET-h/week)**	15.7 (9.4, 22.1)	16.1 (10.4, 22.6)	15.5 (11.1, 22.6)	0.3	9.7 (5.3, 17.3)	10.7 (6.4, 16.5)	11.0 (7.1, 16.4)	0.4
**Energy intake (kcal/d)**	1712.2 (1482.3, 2031.6)	1715.7 (1453.7, 2011.6)	1799.0 (1442.4, 2083.9)	0.8	2029.0 (1676.6, 2359.0)	2085.6 (1769.6, 2493.5)	2155.3 (1715.2, 2570.7)	0.08
**NSD (7-9h/n, n (%))**	415 (61.8)	621 (63.5)	113 (53.3)	0.1	275 (63.1)	425 (62.5)	94 (59.5)	0.01
**BMI (kg/m**^**2**^**)**	23.9 (21.7, 26.1)	23.2 (21.1, 25.3)	23.6 (22.0, 26.4)	0.002	24.4 (22.2, 26.8)	24.2 (22.1, 26.0)	24.4 (22.7, 26.9)	0.07
**WC (cm)**	85.4 (78.1, 91.8)	83.7 (77.2, 89.9)	83.5 (78.8, 92.5)	0.02	86.9 (80.8, 93.1)	86.4 (80.9, 92.2)	89.0 (83.5, 94.7)	0.02
**BF (%)**	34.1 (31.6, 36.4)	33.4 (30.8, 35.6)	33.5 (31.1, 36.2)	0.004	22.6 (19.8, 24.9)	22.3 (19.8, 24.4)	23.1 (20.8, 25.6)	0.02
**FMI (kg/m**^**2**^**)**	8.2 (6.9, 9.4)	7.7 (6.4, 9.0)	7.9 (6.9, 9.6)	0.003	5.6 (4.4, 6.6)	5.4 (4.3, 6.2)	5.6 (4.8, 6.9)	0.04
**Overweight**^**d**^	254 (37.9)	274 (28.0)	75 (35.4)	0.003	198 (45.4)	258 (37.9)	70 (44.3)	0.1
**Central Obesity**^**e**^	465 (69.3)	638 (65.2)	147 (69.3)	0.3	170 (39.0)	233 (34.3)	70 (44.3)	0.1

Values are median (25th percentile, 75th percentile) or frequencies. Test for difference between groups of MND was performed by using Wilcoxon rank-sum tests for non-normally distributed continuous variables, chi-square test for categorical variables.

MND = Midday Nap Duration; MVPA = Moderate to Vigorous Physical Activity; NSD = Nocturnal Sleep Duration; BMI = Body Mass Index; WC = Waist Circumference; BF = Percentage Body fat; FMI = Fat Mass Index.

^a^ MND was grouped refer the previous study [21].

^b^ School education at least 12 years.

^c^ MVPA energy expenditure, energy expended on moderate-to-vigorous physical activities (MET-hour/week) [29].

^d^ Overweight defined according to the WHO cut-offs for adults[22], BMI ≥25kg/m^2^.

^e^ Central obesity defined according to the IDF[23], WC ≥80 cm for women, ≥90 cm for men.

The associations of NSD with body composition are displayed in **Table 4**. Among men, multiple linear regression analysis showed that NSD was inversely related to BMI (P = 0.0007), WC (P = 0.0002), %BF (P = 0.0001) and FMI (P = 0.0002) after adjustment for age, marital status and education level (model 1). Then adjusted for MND (model 2) or including additional adjustment for EI and MVPA (model 3) did not materially change these inverse associations. No association between NSD with body composition were observed in women (all P > 0.1).

**Table 4:** Indicators of body mass index, waist circumference, percentage body fat and fat mass index by levels of nocturnal sleep duration (n = 3135).

	Nocturnal Sleep Duration^a^ (h/night)			
	< 7 (n = 919)	7~9 (n = 1942)	> 9 (n = 274)	P for trend
**Female**				
**Body mass index (kg/m**^**2**^**)**				
Model 1	23.04 (22.71, 23.38)	23.19 (22.93, 22.45)	22.56 (21.93, 23.20)	0.2
Model 2	23.10 (22.75, 23.45)	23.24 (22.95, 23.54)	22.58 (21.95, 23.23)	0.1
Model 3	23.11 (22.76, 23.47)	23.25 (22.95, 23.54)	22.59 (21.95, 23.24)	0.1
**Waist circumference (cm)**				
Model 1	82.93 (82.00, 83.87)	83.39 (82.67, 84.12)	82.35 (80.56, 84.17)	0.4
Model 2	83.04 (82.05, 84.04)	83.50 (82.68, 84.33)	82.39 (80.58, 84.24)	0.4
Model 3	83.03 (82.04, 84.04)	83.50 (82.68, 84.33)	82.37 (80.56, 84.22)	0.4
**Percentage body fat (%)**				
Model 1	32.72 (32.41, 33.05)	32.89 (32.65, 33.14)	32.44 (31.84, 33.06)	0.3
Model 2	32.76 (32.42, 33.10)	32.92 (32.64, 33.21)	32.45 (31.83, 33.08)	0.3
Model 3	32.77 (32.43, 33.11)	32.93 (32.64, 33.21)	32.45 (31.83, 33.08)	0.3
**Fat mass index (kg/m**^**2**^**)**				
Model 1	7.54 (7.36, 7.72)	7.63 (7.49, 7.77)	7.32 (6.99, 7.66)	0.2
Model 2	7.57 (7.38, 7.76)	7.65 (7.50, 7.81)	7.33 (6.99, 7.68)	0.2
Model 3	7.57 (7.38, 7.77)	7.65 (7.50, 7.82)	7.33 (6.99, 7.68)	0.2
**Male**				
**Body mass index (kg/m**^**2**^**)**				
Model 1	24.03 (23.56, 24.50)	23.83 (23.49, 24.17)	22.60 (21.96, 23.26)	0.0007
Model 2	24.20 (23.71, 24.69)	24.00 (23.64, 24.36)	22.74 (22.08, 23.42)	0.0006
Model 3	24.19 (23.71, 24.68)	23.98 (23.61, 24.34)	22.75 (22.09, 23.43)	0.0007
**Waist circumference (cm)**				
Model 1	86.24 (84.95, 87.55)	85.29 (84.37, 86.28)	81.81 (80.02, 83.64)	0.0002
Model 2	86.78 (85.45, 88.13)	85.85 (84.85, 86.85)	82.30 (80.46, 84.18)	0.0002
Model 3	86.81 (85.48, 88.17)	85.89 (84.90, 86.90)	82.32 (80.48, 84.20)	0.0001
**Percentage body fat (%)**				
Model 1	21.63 (21.10, 22.17)	21.27 (20.89, 21.65)	19.82 (19.11, 20.56)	0.0001
Model 2	21.82 (21.05, 21.88)	21.46 (21.05, 21.88)	19.98 (19.25, 20.74)	0.0001
Model 3	21.83 (21.28, 22.40)	21.47 (21.06, 21.89)	19.99 (19.26, 20.75)	0.0001
**Fat mass index (kg/m**^**2**^**)**				
Model 1	5.20 (4.98, 5.43)	5.07 (4.91, 5.23)	4.48 (4.21, 4.77)	0.0002
Model 2	5.28 (5.05, 5.52)	5.15 (4.98, 5.32)	4.54 (4.26, 4.84)	0.0002
Model 3	5.28 (5.05, 5.52)	5.15 (4.98, 5.32)	4.55 (4.26, 4.85)	0.0002

Values are least-squares means (95% confidence interval) from models. Linear trends (P for trend) were obtained with nocturnal sleep duration as continuous variables.

Model 1 was adjusted for age, marital status and education level.

Model 2 was adjusted for midday nap duration in addition to model 1.

Model 3 was adjusted for MVPA and EI in addition to model 2.

^a^ Nocturnal sleep duration was grouped according to the National Sleep Foundation [20].

An association between MND and body composition was observed in logistic regression analysis (Table 5). In basic model, the results indicated that people napped 0.1-1h compared to people with no midday nap was significantly associated with prevalence of overweight in women (OR: 0.73, 95%CI: 0.55–0.96) but not in men (OR: 0.76, 95%CI: 0.56–1.05). After adjustment for all potential confounders, compared to the subjects in the no midday nap group, the subjects who napped 0.1–1 hour were independently associated with a less prevalence of overweight in both women (OR: 0.72, 95%CI: 0.55–0.95) and men (OR: 0.71, 95%CI: 0.52–0.98). We found no association of MND with central obesity in the present analysis (Table 6).

**Table 5:** Multiple logistic regression OR and 95% CI for the association of groups of midday nap duration with overweight (n = 3135).

Overweight (yes or no)		Midday Nap Duration^a^ (h)				
		0	0.1–1		> 1	
			OR (95% CI)	P	OR (95% CI)	P
**Female**	**Model 1**	Reference	0.73 (0.55–0.96)	0.02	0.85 (0.55–1.29)	0.4
	**Model 2**	Reference	0.73 (0.55–0.96)	0.02	0.86 (0.56–1.31)	0.5
	**Model 3**	Reference	0.72 (0.55–0.95)	0.02	0.87 (0.57–1.32)	0.5
**Male**	**Model 1**	Reference	0.76 (0.56–1.05)	0.09	0.92 (0.58–1.48)	0.7
	**Model 2**	Reference	0.73 (0.53–1.00)	0.05	0.87 (0.54–1.40)	0.6
	**Model 3**	Reference	0.71 (0.52–0.98)	0.04	0.86 (0.53–1.38)	0.5

Values are OR with 95% CI for overweight by groups of MND.

OR = odds ratio; CI = confidence interval.

Model 1 was adjusted for age, marital status and education level.

Model 2 was adjusted for nocturnal sleep duration in addition to model 1.

Model 3 was adjusted for MVPA and EI in addition to model 2.

^a^ MND was grouped refer the previous study [21].

**Table 6:** Multiple logistic regression OR and 95% CI for the association of groups of midday nap duration with central obesity(n = 3135).

Central Obesity (yes or no)		Midday Nap Duration^a^ (h)				
		0	0.1–1		> 1	
			OR (95% CI)	P	OR (95% CI)	P
**Female**	**Model 1**	Reference	1.01 (0.76–1.35)	0.9	1.09 (0.69–1.74)	0.3
	**Model 2**	Reference	1.02 (0.76–1.36)	0.9	1.09 (0.69–1.75)	0.9
	**Model 3**	Reference	1.02 (0.76–1.36)	0.9	1.10 (0.69–1.76)	0.7
**Male**	**Model 1**	Reference	0.83 (0.60–1.14)	0.2	1.21 (0.76–1.95)	0.4
	**Model 2**	Reference	0.80 (0.58–1.10)	0.2	1.15 (0.71–1.85)	0.6
	**Model 3**	Reference	0.80 (0.57–1.10)	0.2	1.14 (0.71–1.84)	0.6

Values are OR with 95% CI for central obesity by groups of MND.

OR = odds ratio; CI = confidence interval.

Model 1 was adjusted for age, marital status and education level.

Model 2 was adjusted for nocturnal sleep duration in addition to model 1.

Model 3 was adjusted for MVPA and EI in addition to model 2.

^a^ MND was grouped refer the previous study [21].

## Discussion

This study has demonstrated an inverse association between NSD and body composition in men, but not in women; additionally, midday nap <1h was significantly related with the lower prevalence of overweight in both Chinese females and males.

Our results demonstrated that NSD was inversely related to body composition among men, which is consistent with previous studies including Chinese[13] and Western adults[12], but some other studies have the conflicting conclusion[14,15]. The differences in characteristics of participants, adjustment factors and measurement of sleep duration may partially account for the observed differences among studies. We were interested in exploring gender-specific results, although gender differences did not produce statistical tests for interaction in this study, as can be seen from the characteristics of participants, there were significant differences in BF and FMI between men and women. We also observed significant differences in EI and PA between different genders, however, adjusted them did not change the results. The potential mechanism of obesity caused by short sleep duration have been examined, short-term sleep restriction affected the circulating concentrations of appetite-related hormones (leptin and ghrelin) [30], the average age of women in present study was around menopause, hormone changes during menopause may have been affected this mechanism. It may explain the gender differences in this study.

Another finding of this study is that MND was also related to overweight but not central obesity. The result is consistent with the large Mediterranean cohort study [17]. In contrast, one study evaluating the relation between MND and the prevalence metabolic syndrome in Chinese adults [31] found that the prevalence of central obesity was higher in people who napped >1h than people in no midday napping group. Such inconsistent result may be due to not considering variables such as EI use being possible related to body composition and different diagnosed criteria of metabolic syndrome have different definitions of central obesity. The mechanisms for the association between siesta and body composition were not clear. However, other benefits of proper napping to people were reported. Luo, Z. et al. analyzed four males and four females aged 27–35 years with emotion spectrum and found that after siesta, the subjects were significantly more joyful and relaxed, indicating that a short nap can improve mood [32]. And a prospective cohort study reported that siesta was inversely associated with coronary mortality [33].

Physical activity and EI may be the mediator in sleep-obesity association. After adding them into model, the statistical association between NSD and body composition has not been attenuated which reflects they couldn’t fully explain the association, maybe there was an alternative pathway between NSD and body composition. People who sleep less may have more stress than those who sleep much. This may also affect the metabolism by activating the adrenal cortical pathway[34]. The link between sleep duration and the incidence of diabetes have been determined in several researches[35], short sleepers may lead to obesity by altering glucose metabolism. While the statistical relationship between MND and overweight has become significant after adjusted physical activity and EI, that suggests physical activity and EI as confounding factors have weaken the relationship between MND and overweight.

Several strengths of our study deserve mentioning: we evaluate the association between NSD and MND with body composition. In particular, we performed detailed assessment of sleep duration by trained investigators in face-to-face interviews, and we considered the difference in NSD on weekends and workdays. Moreover, we stratified gender to observe the gender-specific on relationship between sleep duration and body composition. A further strength lies in the adjustment for a number of confounding factors that potentially affected the association between sleep duration and body composition, particularly socioeconomic indices, sleep variable, physical activity and EI.

Our study also has limitations. Firstly, due to the cross-sectional design of our analysis, causal relationships could not be established. Secondly, subjective sleep data may be biased because adults tend to overreport their actual sleep time [36] and the question approach does not provide information on sleep quality. Thirdly, using predictive equations to predict %BF and FMI will produce prediction errors, in despite of we have used standardized procedures to measure them.

## Conclusions

Our study illustrates that the NSD and MND were of relevance for body composition among Southwest Chinese adults. The association between NSD and body composition was inverse in men, but not in women. Additionally, rational midday nap might be an independent protective factor for overweight in adults;

## Abbreviations

EI: energy intake
NSD: nocturnal sleep duration
MND: midday nap duration
